# A Willingness-to-Pay Associated Right Prefrontal Activation During a Single, Real Use of Lipsticks as Assessed Using Functional Near-Infrared Spectroscopy

**DOI:** 10.3389/fnrgo.2021.731160

**Published:** 2021-11-26

**Authors:** Kazue Hirabayashi, Tatsuya Tokuda, Tomomi Nishinuma, Keith Kawabata Duncan, Keiko Tagai, Ippeita Dan

**Affiliations:** ^1^MIRAI Technology Institute, Shiseido Co., Ltd., Yokohama, Japan; ^2^Applied Cognitive Neuroscience Laboratory, Chuo University, Tokyo, Japan

**Keywords:** functional near-infrared spectroscopy (fNIRS), lipstick, consumer neuroscience, dorsolateral prefrontal cortex (dlPFC), prefrontal cortex, willingness-to-pay (WTP), cosmetics

## Abstract

Understanding consumer preferences and behavior is a major goal of consumer-oriented companies. The application of neuroscience to this goal is a promising avenue for companies. Previously, we observed a positive correlation during actual cosmetic use between the right dorsolateral prefrontal cortex (dlPFC) activity, measured by functional near-infrared spectroscopy (fNIRS), and the associated willingness-to-pay (WTP) values. However, we were unable to find any consistent group differences in the right dlPFC between different powdery foundations. Thus, the main objective of this study was to replicate the previous study and in addition, we aimed to refine the method of the previous study to increase the chance that a difference in valuation between different products can be detected. Twenty-five frequent lipstick using females were asked to apply six different lipsticks to their lips and to record how much they were willing to pay. To maximize the variation of the subjective experience of the products and the associated brain activity, the most preferred color lipstick and a less preferred color lipstick were chosen for each participant, and each color of lipstick had three different textures (*Lo, Mid*, and *Hi*). The time series was analyzed with the general linear model (GLM) and the correlation between the right dlPFC beta scores for the lipsticks and their respective WTP values conducted for each participant. This revealed a significant positive correlation and replicated our previous study. Surprisingly, the lipstick color and the texture manipulations did not result in any consistent differences in WTP and similarly no consistent group differences in brain activations. This study replicates our previous study extending it to a different type of cosmetic. The right dlPFC activity during the use of cosmetics may be a potential brain-based personalization or product selection process biomarker.

## Introduction

A major goal of any consumer-oriented company is to understand consumer preferences and behavior. An important step to achieve this goal was made by the application of psychological knowledge to economic human decision-making (Kahneman and Tversky, 1979). Furthermore, the application of the neuroscientific approach, best exemplified by neuroimaging methods, has been applied commercially to complement traditional methods of consumer research. Several regions of the prefrontal cortex (PFC) may play a role in consumer assessment of products based on perceived benefits and cost (Solnais et al., 2013).

The expectation is that the differences in brain activity can reveal more information about the responses of consumers to the products. This is because the brain-derived measurements of consumer attitudes are not always the same as subjective choice preferences (Ramsøy et al., 2019), with one possible reason being that brain activity is more likely to be free of biases. This in turn may allow more accurate testing of products and prototypes even with smaller sample sizes relative to traditional self-report. Thus, applying consumer neuroscience to existing product development, marketing or selling strategies will help companies more deeply understand the decision-making of their customers (Berčík et al., 2016), and therefore, provide them with superior products and services.

Functional near-infrared spectroscopy (fNIRS) is a promising neuroimaging method for consumer neuroscience as it can be used to investigate brain activity in naturalistic and realistic environments with low cost. A number of studies have used fNIRS to measure brain activity during consumer behavior. For example, fNIRS has been used to assess the decision-making behavior of consumers when they imagine shopping (Krampe et al., 2018) and when they evaluate specific food labels of brands using mobile fNIRS (Meyerding and Mehlhose, 2020). In terms of measuring the brain responses of consumers to products using fNIRS, one promising area is the right dorsolateral prefrontal cortex (dlPFC) (Plassmann et al., 2007). In a functional MRI (fMRI) study, Plassmann et al. (2007), in addition to the medial occipitotemporal cortex (mOFC), activity in the right dlPFC was found to correlate with willingness-to-pay (WTP), the maximum amount of money a person is willing to part with obtain a product. Unlike the mOFC, activity in the dlPFC can be readily measured using fNIRS.

As a first step in developing the use of the dlPFC activity as a potential biomarker of consumer preferences and future behavior, we have investigated whether brain activity measured during a single, real use of cosmetics contains meaningful information. This first step is important because the brain activity recorded by fNIRS is noisy especially during a real-world activity, such as applying cosmetics. We previously observed that an intrasubject correlation between activation in a right dlPFC of a participant correlated positively with their WTP for a cosmetic product (Kawabata Duncan et al., 2019). Specifically, participants applied six powdery foundations which varied in quality to their faces, while brain activity was measured using fNIRS. The right dlPFC is a large brain area, and it is not clear which fNIRS channel corresponds to the area. Therefore, virtual registration allows the registration of fNIRS data into Montreal Neurological Institute (MNI) standard brain space (Tsuzuki et al., 2007). Once registered to MNI space, it is possible to identify the channel closest to the peak identified by Plassmann et al. (2007). The activity of participants in this channel for the different foundations was correlated with their WTP. Interestingly, this correlation was only found in higher frequency users of powdery foundation, suggesting a role of experience in evaluating the product.

The right dlPFC has varied roles, including cognitive control, working memory, and emotion regulation (Duncan and Owen, 2000; Curtis and D'Esposito, 2003; Golkar et al., 2012). This higher order cognitive processing can be integrated with reward signals in other areas, such as the mOFC, to improve decision-making (Hare et al., 2009). To understand why such a correlation may occur, it is helpful to understand the experience of using a cosmetic, such as foundation or lipstick. The dominant sensation is experienced during the application of cosmetic changes over the period of application (Boinbaser et al., 2015). Taking lipsticks as an example, at first, the user may notice the color of the lipstick, then its fragrance, softness, and finally the overall visual impression of the finished lips. This means that the evaluation of a cosmetic product, such as lipstick, by a consumer is not instantaneous but relies on monitoring the visual effects and feelings of use which change dynamically during the application period and integrating this information to form a final unified evaluation. If the color of the lipstick is poor, there is little need to continue the evaluation, resulting in less cognitive processing, and thus less activity in the right dlPFC over the lipstick application. In other words, we expect large activation occurs when the evaluation by the subject of preferred products engages complex and analytical decision-making process following the temporal sensory perceptions during using cosmetics.

However, due to the limitation in the number of times the face can be cleansed of cosmetics, the positive correlation between the right dlPFC activity and WTP observed in our previous study was based on six trials (Kawabata Duncan et al., 2019). Therefore, the main objective of this study was to replicate the previous study to rule out the possibility that the previous result was obtained through chance. In addition, we aimed to refine the method of the previous study to increase the chance that a difference in product valuation between different products can be detected. This is important because to be an effective biomarker of consumer preferences, etc., the right dlPFC activity needs to differentiate between different products. This refinement involved the following: first, a chin rest was used to reduce head motion-associated noise. Second, we used lipstick as the test product because this allowed us to manipulate both color and quality, to maximize the difference between the least preferred and most preferred products.

## Methods

### Participants

Twenty-five right-handed female participants (average age: 29.6, SD: 2.9, range: 25–35) participated after giving their informed consent. All participants reported that they were able to communicate and read in Japanese and/or English. In addition, they used lipstick at least 5 times per week. The experiment was conducted in the Applied Cognitive Neuroscience Laboratory in Chuo University, Bunkyo-ku, Tokyo under the approval of the local ethics committees of both Chuo University and Shiseido Co., Ltd.

### Lipsticks

Three levels of qualities (*High, mid*, and *lo*) of lipsticks were prepared in six colors for this test. The quality levels of the lipsticks were determined based on the results of the product evaluation test in the United States of America organized by Shiseido Co., Ltd. *High* and *mid* qualities of lipsticks were products of the brand *MAQuillAGE*. The *lo* quality lipstick was a prototype sample, which was hard and had a matt texture (see Table 1A). Six different colors were chosen from an internal color preferences guide. Before the beginning of the test, participants were asked to place six colors of lipsticks in order of their preferences. For each subject, their most preferred color was selected as their subject-specific “*like*” color, and the third least preferred lipstick was selected as their “*less like*” color. This was to avoid the possibility that participants could give a WTP of zero for lipsticks which were a color they did not like at all. Note that the color condition does not mean a specific color, and thus colors liked or less liked differed depending on the participants. Accordingly, in the test, participants tested six samples; two colors (*like* and *less like*) in three qualities (*High, mid*, and *lo*) (see Table 1). Averaging the responses to “*liked*” thus represents the brain and subjective responses to “*liked*” lipsticks, regardless of the color. In the same way, averaging responses to “*less liked*” represents the brain and subjective responses to “*less liked*” lipsticks. The goal was to create a range of subjective experiences, which are meaningful when averaged across participants, with the aim of maximizing the chance of finding a difference between brain responses to the lipsticks and/or a correlation between WTP and brain responses.

**Table 1:** Lipstick samples. **(A)** Lipsticks product details. **(B)** Lipstick ID.

**Quality**	**Product**
**(A)**	
High (J)	MAQuillAGE Dramatic Rouge P
Mid (C)	MAQuillAGE Dramatic Rouge
Low (G)	Prototype (matt texture)
**Lipstick ID**	
**(B)**	
**SJ** **=** most favorite color and *high* quality	**KJ** **=** less liked color and *high* quality
**SC** **=** most favorite color and *mid* quality	**KC** **=** less liked color and *mid* quality
**SG** **=** most favorite color and *high* quality	**KG** **=** less liked color and *low* quality

*3 different lipsticks for this test are based on the product texture*.

*The colors of S and K are selected from six colors based on color preference order of each participant. These IDs are the same for each subject, but the colors are subject specific*.

### fNIRS Experimental Design

**Figure 1:**
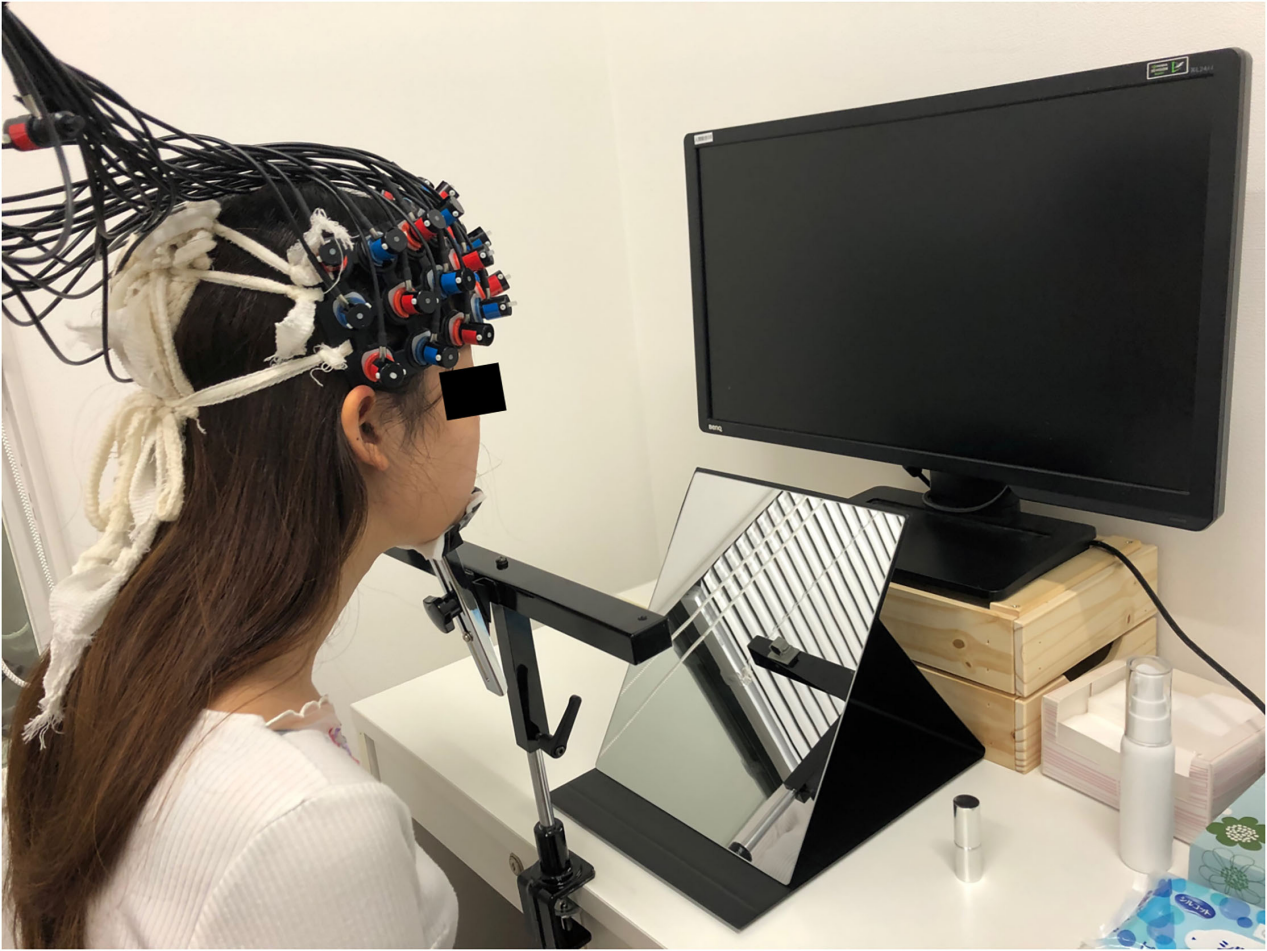
Experiment image. Participants sat in front of the mirror and the PC monitor which displayed the visual information. Head motion can cause a deterioration in the signal-to-noise ratio in the fNIRS data, therefore a chin rest was used to reduce head motion during using lipsticks. fNIRS, functional near-infrared spectroscopy.

Before the experiment began, participants received a written and verbal explanation of the experiment and that the experiment was conditional on their informed consent, where there would be no disadvantage conferred upon them should they choose to not participate. The participants were informed that they were required to apply lipstick and then type their willingness to pay for the product using a keyboard. We explained that WTP does not mean how much you think the price should be or how much you want it to be, but rather the maximum amount of money you would be willing to spend to get the lipstick. In addition, we asked them to take both the quality and color into consideration. If the quality and color were such that there would be no possibility of purchase, they could respond 0 for WTP. There was no upper limit for their WTP. Moreover, they were asked to use the same currency throughout the experiment. Each lipstick was applied to only one-half of the lips (left or right) to reduce the number of times the lips needed to be cleansed of lipstick to minimize the burden on the delicate skin of the lips. Participants sat in front of a mirror and a PC monitor which displayed the visual information. Head motion can cause a deterioration in the signal-to-noise ratio in the fNIRS data. Since it can be challenging to hold one's head still while at the same time applying cosmetics to the face, a chin rest was used. This stabilized the head and reduced the amount of movement the participant could make with their head without undue restraint (Figure 1).

**Figure 2:**
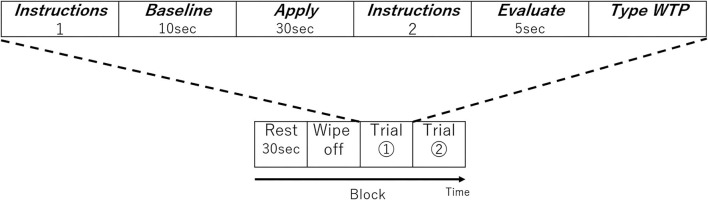
One block of the experimental design. Each block consisted of two trials. The order of lipstick and the side to which they were applied were randomized.

In the fNIRS experimental design, one block was composed of four periods; *Rest, Wash, Trial 1*, and *Trial 2*. Before the first trial, the participant went through a single block without applying any lipstick as a practice session to get used to the process. In the *Rest* period, they kept still for 30 s (Figure 2). In the *Wash* period, they removed the lipsticks on their lips using a cotton pad with a cosmetic. A facial emulsion (*Elixir Superieur Lift Moist Emulsion*) was selected as a low skin irritation cosmetic to remove the lipstick. Once the participant finished cleansing their lips, the first set of instructions were displayed on the PC monitor informing the participant which lipstick sample (SJ, SC, SG, KJ, KC, or KG; see Table 1B) they would use next and which side of the lip (left or right side) they would apply the lipstick in this session (*Instructions 1* in Figure 2). As it was not practical to fully counterbalance both lipstick sample and lip side (six lipsticks and two lipstick sides), the order of lipsticks and the combination of lipstick sample and lip side were randomized for each participant. After the participant received the lipstick and was ready to start, 10 s of baseline brain activity were recorded (*Baseline* in Figure 2). Then, an auditory cue informed the participant that they should begin applying the lipstick and keep applying the lipstick for 30 s (*Apply* in Figure 2) until another auditory cue informed them to stop. The second set of instructions (*Instructions 2* in Figure 2) was then displayed on the PC screen for the participant to press the Enter key when ready and the monitor displayed a blank white screen for 5 s and the participant should think about how much they would be willing to pay for the lipstick they have just used (*Evaluation* in Figure 2). After this 5 s elapsed, on the PC screen, the following text appeared, “Please type how much you are willing to pay.” Then they typed their WTP with a keyboard and pressed the Enter key (*Type WTP* in Figure 2). The participant was able to correct any typing mistakes they made using the Backspace key. The entry of the WTP was terminated by the participant pressing the Enter key. Therefore, a single trial comprised the lipstick application to the half side of the upper and lower lip, evaluation, and entering of WTP of a single lipstick. In the next trial, the procedure started from *Instructions 1*. The monitor displayed a different lipstick sample from the first trial and the other side of the upper and lower lip to apply the lipstick. Then the trial proceeded in the same way as the first trial. The visual and auditory information were presented using E-Prime (E-Prime, Psychology Software Tools, Inc., Sharpsburg, PA, USA).

### Functional Near-Infrared Spectroscopy

In the experiment, hemodynamic responses of the brain were measured with a multichannel fNIRS optical topography system ETG-4000 (Hitachi Medical Corporation, Kashiwa, Japan) using dual wavelengths of near-infrared light (695 and 830 nm) at a 10 Hz sampling rate. The optical data were analyzed based on the modified Beer-Lambert Law (Cope et al., 1988). Accordingly, signals reflecting concentration changes of the oxygenated hemoglobin (oxy-Hb) and deoxygenated hemoglobin (deoxy-Hb) were obtained in units of millimolar·millimeter (mM·mm) (Maki et al., 1995). For our analysis, we focused on oxy-Hb signals since it was shown to be more reliable than the deoxy-Hb signal (Dravida et al., 2018) and only the oxy-Hb showed a correlation with behavior in our previous research (Kawabata Duncan et al., 2019).

### Spatial Registration

**Figure 3:**
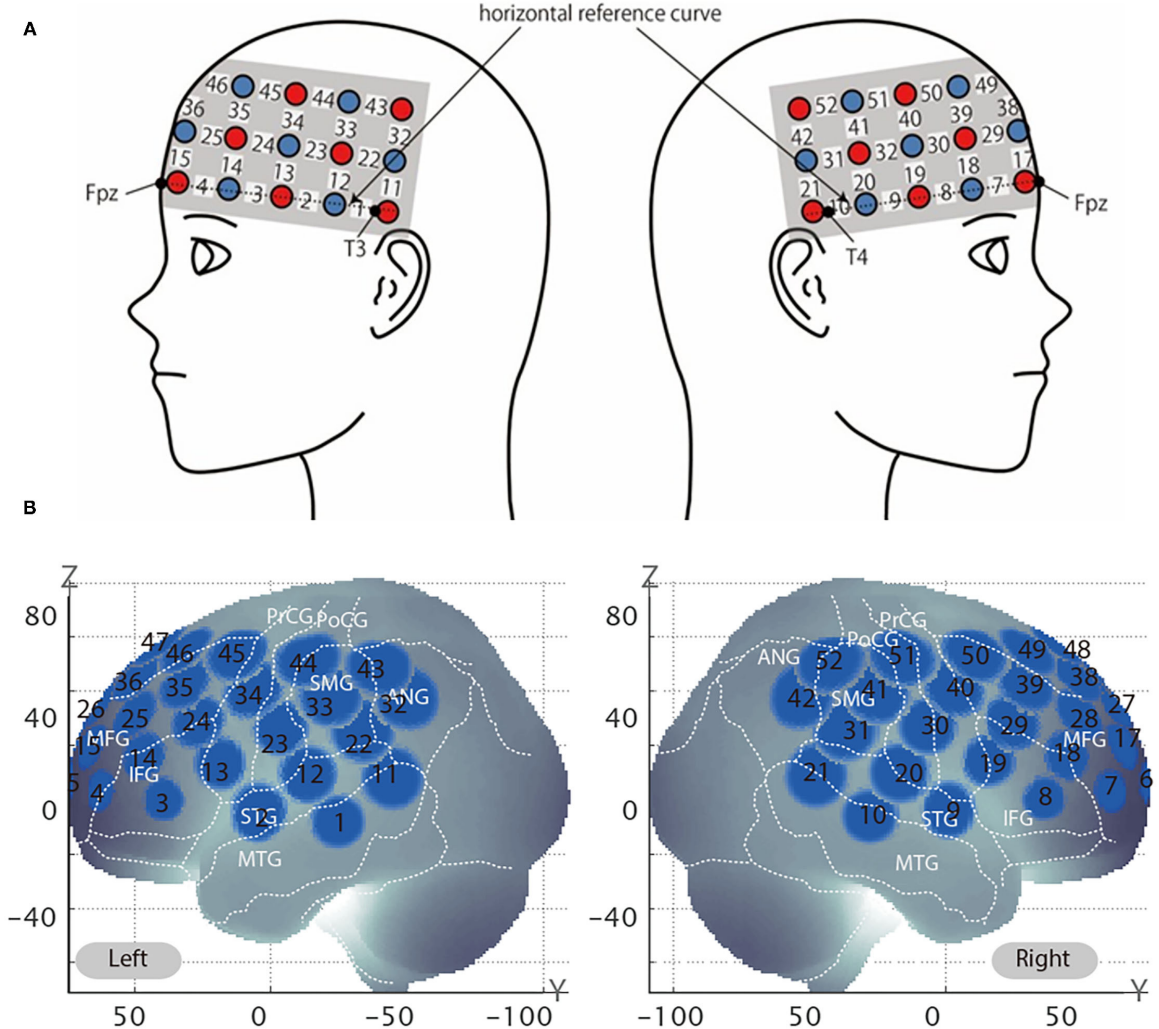
Virtual estimated channel positions. **(A)** Spatial profiles of functional near-infrared spectroscopy (fNIRS) channels. The upper panel shows left- and right-side views of the probe arrangements are shown with fNIRS channel orientation. Detectors are indicated with dark circles, illuminators with light circles, and channels with white squares. Corresponding channel numbers are shown in black. **(B)** The estimated channel locations on the brain for both left and right side views are shown. The circles indicate the spatial variability associated with the estimation exhibited in the MNI space.

After all the testing blocks, the position of each probe was recorded using a 3D digitizer (POLHEMUS, Patriot). We used a 3 × 11 multichannel probe holder consisting of 17 illuminating and 16 detecting probes arranged alternately at an interprobe distance of 3 cm (Figure 3A). We set Fpz at the middle point of the lowest row of the probe holder. The channel positions were registered to the MNI standard brain space (Brett et al., 2002) based on the virtual registration method (Okamoto et al., 2004; Tsuzuki et al., 2007). Simulated 52 channel positions were visualized on the reference scalp from the probe records are shown in Figure 3B. The lowest line of the probes was placed over the horizontal reference curve, which includes T4, Fpz, and T3, and we set the probe holders placed along with the location of Fpz, T3, and T4 as Figure 3B (Tsuzuki et al., 2007). We defined the midpoint of a pair of illuminating and detecting probes as a channel location. Estimated locations were anatomically labeled using a MATLAB function which reads anatomical labeling information coded in a macroanatomical brain atlas, Automated Anatomical Labeling (AAL) (Tzourio-Mazoyer et al., 2002), LBPA40 (Shattuck et al., 2008), and Brodmann's atlas (Rorden and Brett, 2000).

The right dlPFC corresponds to mainly Brodmann areas 9 and 46 (Rajkowska and Goldman-Rakic, 1995), with several channels potentially giving coverage. Setting the channel of interest to the same channel identified in our previous study (channel 39) may actually result in the analysis of data obtained from a different area of the brain, because the coverage of the channels may differ depending on various factors, such as the head shape of the participants. In the previous test, the participants were Japanese whereas in the current experiment, the participants self-identified as Caucasian, and there are significant head shape differences between the two groups (Ball et al., 2010). Therefore, we used the same method to identify the channel of interest in the current experiment as we did previously. Specifically, the channel closest to the peak (*x* = 44, *y* = 44, and *z* = 18) previously reported correlating with WTP by Plassmann et al. (2007) was identified by calculating the Euclidean distance from the said peak to the MNI coordinates of each channel. The channel closest to the right dlPFC was channel 38 (Table 2).

**Table 2:** MNI coordinates.

**Channel**	**x**	**y**	**z**	**SD**	**Anatomy**	**Probability**
38	23.3	55.7	39.3	11.0	9 - Dorsolateral prefrontal cortex 46 - Dorsolateral prefrontal cortex 10 - Frontopolar area	0.80 0.18 0.03

*The MNI coordinates of channel 38 and the probability of the recorded data originating from the listed anatomical locations. MNI, Montreal Neurological Institute*.

### Analysis of fNIRS Data

First, channel data containing unvaried periods exceeding 10% or more of the timeline were excluded. Using Wavelet minimum description length (MDL) detrending algorithm (Jang et al., 2009), we removed global trends due to breathing, cardiac movement, vasomotion, and other experimental artifacts. Then we preprocessed oxy-Hb time-series data for each channel of each participant using MATLAB 2007b (The MathWorks, Inc., Natick, MA, USA) with the tools from Uga et al. (2014) using the adaptive GLM. Relative to averaging fNIRS data across a time interval of interest, the adaptive GLM increases statistical power by incorporating variability by using the temporal information of oxy- and deoxy-Hb signals (Uga et al., 2014). The data were regressed with a linear combination of explanatory variables. The regressors were created by convolving the hemodynamic response function (HRF) shown in Equation 1 with the boxcar function *N*(τ_*p*_, *t*) (Equation 2) (Friston et al., 1998). We set 6 s for the first peak delay, τ_*p*_, as is commonly done and 16 s for the second peak delay, τ_*d*_, was set to 16 s, and *A*, the amplitude ratio between the first and second peak, was set to 6 s. The first and second derivatives were included to eliminate the influence of noise of individual data further.

Equation 1: HRF


(1) $$\begin{array}{c} h\left(\tau_{p},\;t\right)=\frac{\;t^{\tau_{p}}\;e^{-t}}{(\tau_{p})!}\;-\frac{t^{\tau_{p}}{}^{+\tau_{d}}e^{-t}}{A(\tau_{p}+\tau_{d})!} \end{array}$$

Equation 2: Model waveform created by convolving the HRF and boxcar function.


(2) $$\begin{array}{c} f\left(\tau_{p},\;t\right)=h\left(\tau_{p},t\right)*N(\tau_{p,\;}t) \end{array}$$

**Figure 4:**
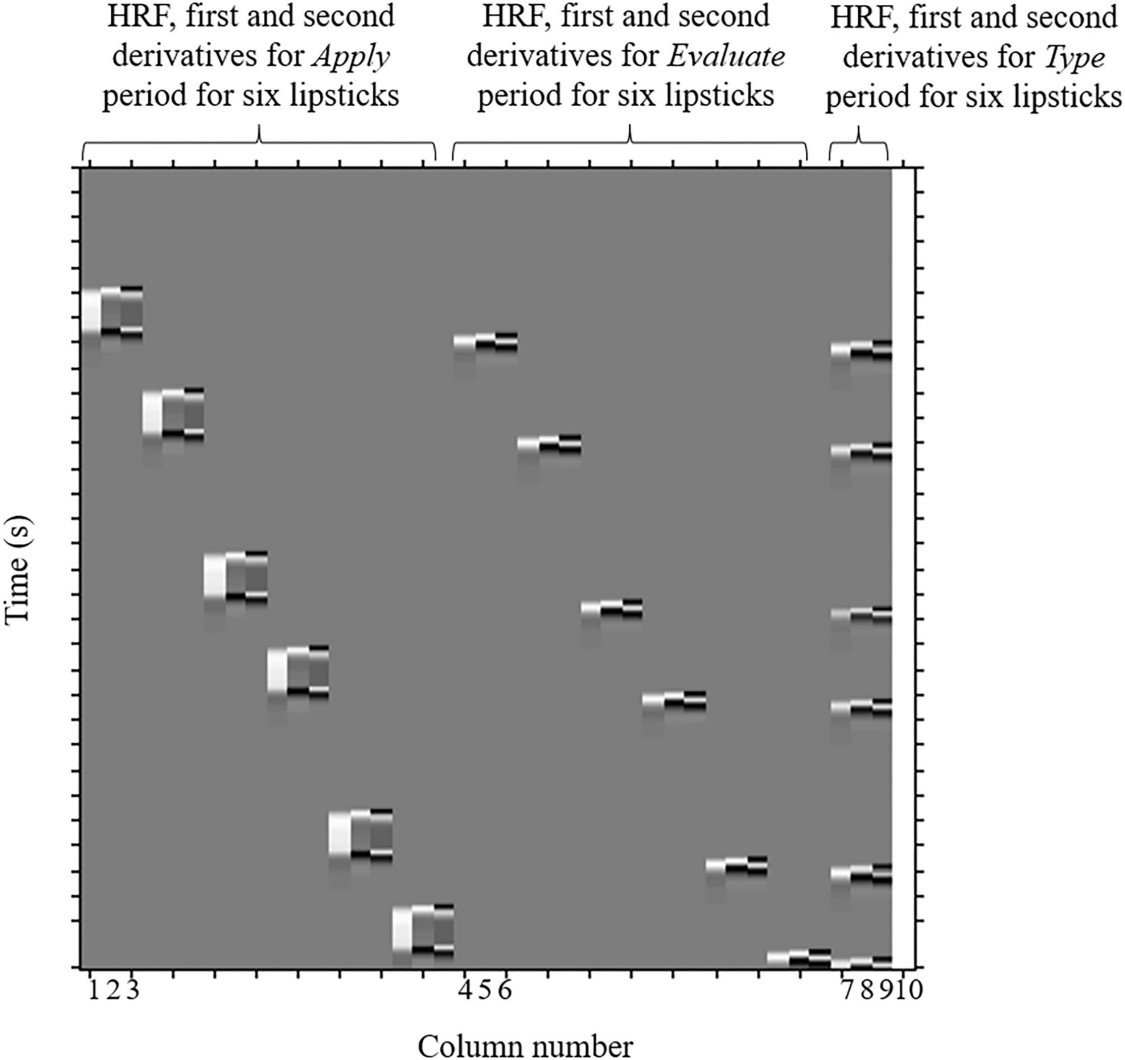
An example design matrix for the GLM model. This is a design matrix from one of the participants. The design matrices of different participants will differ because of the randomization of samples and due to the fact that the duration of certain parts of the experiment differ between subjects (because the duration depends on how long it took the participant to enter their WTP then press enter, and how long it took to cleanse their lips). The first peak delay was set as τ_*p*_ = 6 s and the row number represents the time sequence with time zero being at the top. The columns designated with 1, 2, and 3 indicate the canonical hemodynamic response function (HRF) *f* (τ_*p*_, *t*), the derivatives, and the second derivatives, respectively, for *Apply* period. There were six triplets of the regressors for application, representing six different samples. The columns designated with 4, 5, and 6 indicate those for the *Evaluation* period. There were six triplets of the regressors for evaluation, representing six different samples. The column designated with 7, 8, and 9 indicates those for the *Type WTP* period. The column indicated with 10 indicates the constant. GLM, general linear model; WTP, willingness-to-pay.

The regressors included in the GLM analysis were the 30 s *Apply*, the 5 s *Evaluate*, and the *Type WTP* for each trial. Columns 1–3 in Figure 4, respectively, represent the HRF of the *Apply* period and the first and second derivatives. Columns 4–6, respectively, represent the HRF of the *Evaluate* period and the first and second derivatives. Columns 7–9, respectively, represent the HRF of the *Type WTP* period and the first and second derivatives. Column 10 represents the constant. The β value is used as an estimate of the HRF prediction of the oxy-Hb signal. A total of six β values were calculated for both the *Apply* period (β_*A*1_, β_*A*2_, β_*A*3_, β_*A*4_, β_*A*5_, and β_*A*6_) and the *Evaluate* period (β_*E*1_, β_*E*2_, β_*E*3_, β_*E*4_, β_*E*5_, and β_*E*6_) and a single β value for the *Type WTP* period (β_*type*_), giving a total of 13 β values. β_*A*1_ is the β for the *Apply* period of the first lipstick, β_*E*1_ is the β for the *Evaluate* period of the first lipstick, and so on.

To investigate the relationship between the brain activity of each subject and their WTPs, a group averaged intrasubject correlation was obtained as follows. The Spearman correlation coefficient between the *Apply* period beta values of each channel and the WTP scores for each participant was calculated. Next, the coefficient of each participant was converted into a Z score using Fisher's r-to-z transformation. Then, the average Z score of all participants was calculated and a one-sample *t*-test was conducted to determine whether each channel's mean Z score significantly differed from 0. The group R was calculated from the group Z score using the inverse Fisher transformation. The above process was repeated for the preprocessed deoxy-Hb time-series data, however, only the results for the oxy-Hb time-series data for our channel of interest, channel 38, are described below.

## Results

### Behavioral Performance

**Figure 5:**
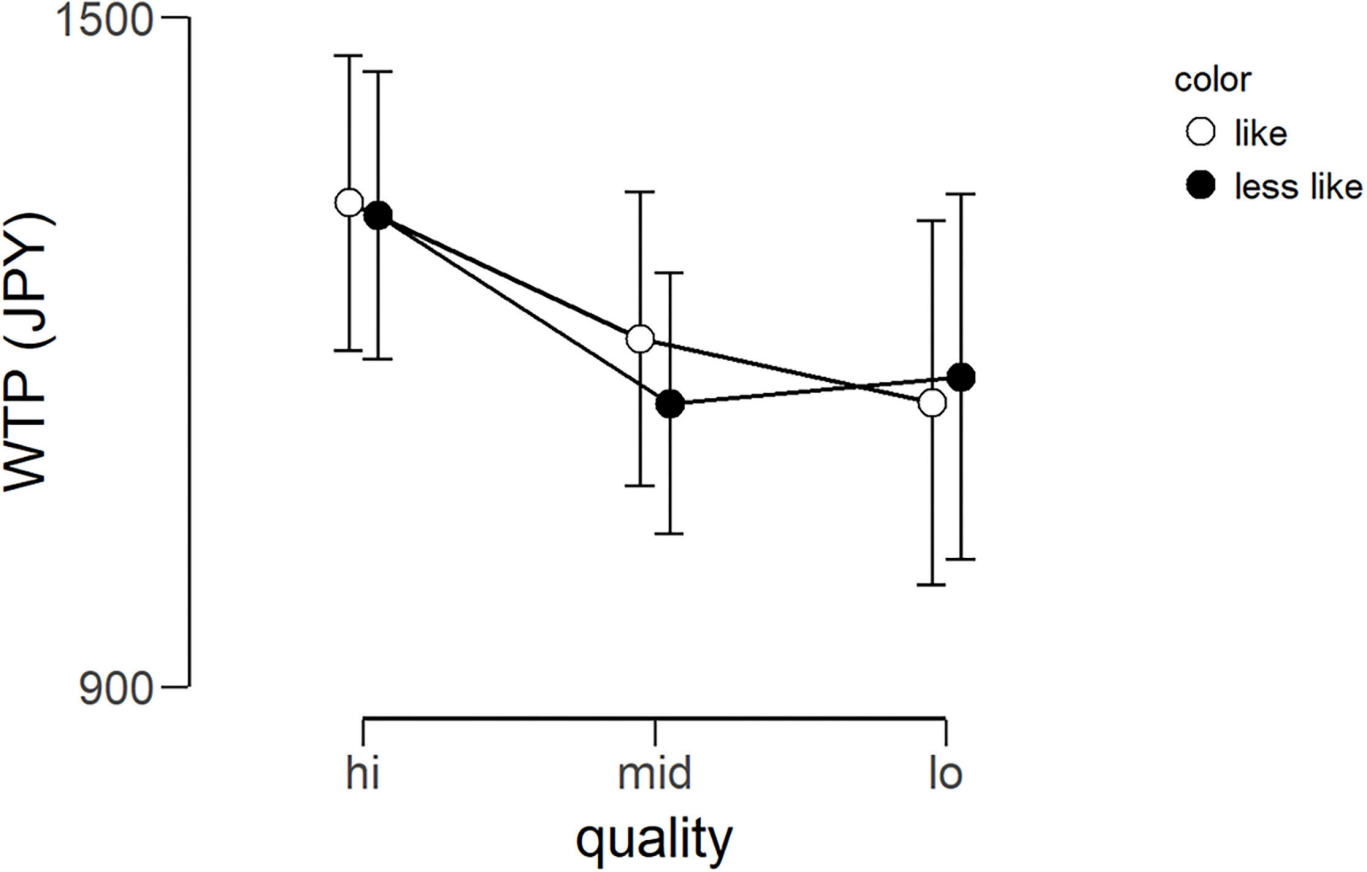
Average WTP (Japanese yen) for six lipsticks. Graph showing the group average WTP scores of the three qualities for the two color conditions (*less like* and *like*). Error bars represent the standard error of the mean. WTP, willingness-to-pay.

The average WTP for each lipstick can be seen in Figure 5. The WTP data were subjected to a 2 × 3 repeated-measures ANOVA with color (*like* and *less like*) and quality (*hi, mid*, and *lo*) as within-subject factors. There was no main effect of color [*F*_(1, 24)_ = 0.019, *p* = 0.809, η*p*^2^ = 0.001], no main effect of quality [*F*_(1.612, 38.680^*^)_ = 0.696, *p* = 0.475, η*p*^2^ = 0.028], and no interaction [*F*_(2, 48)_ = 0.047, *p* = 0.954, η*p*^2^ = 0.002]. In other words, unexpectedly, there was no evidence that the amount of money the participants were willing to pay for the lipsticks was affected by the color or quality. ^*^As Mauchly's test of sphericity indicated that the assumption of sphericity was violated, Greenhouse-Geisser's correction was applied.

### fNIRS Data

**Figure 6:**
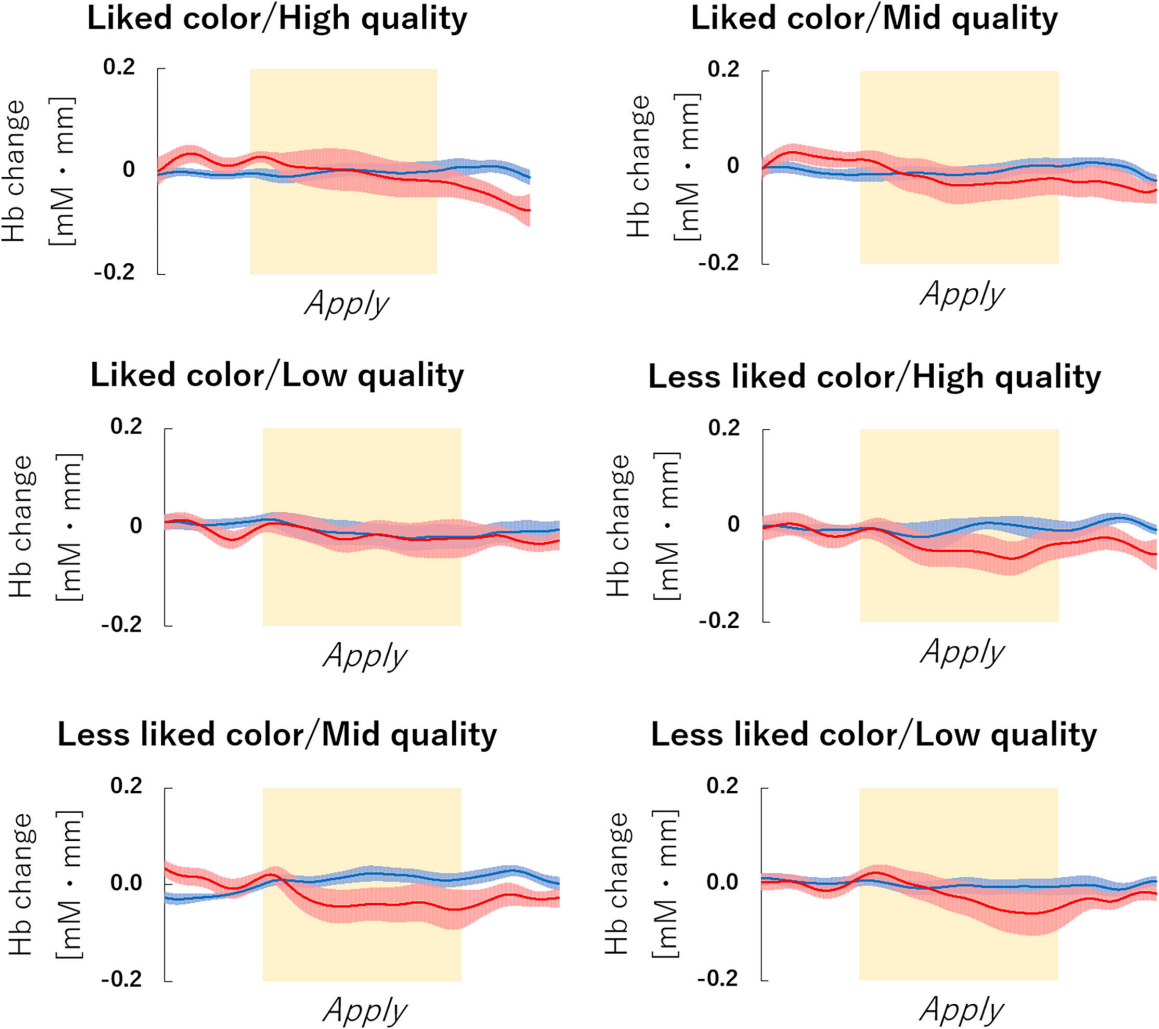
fNIRS Time series data of channel 38 during lipstick application. Graph of the observed time-series data for fNIRS from right hemisphere dorsolateral prefrontal cortex (dlPFC) for six lipsticks, averaged across all subjects. The red lines indicate the observed oxygenated hemoglobin (oxy-Hb) signal and the blue lines indicate deoxygenated hemoglobin (deoxy-Hb) signal. Standard errors are shown as pale red (oxy-Hb) and blue (deoxy-Hb) areas. The yellow highlighted area is the *Apply* period. fNIRS, functional near-infrared spectroscopy.

**Figure 7:**
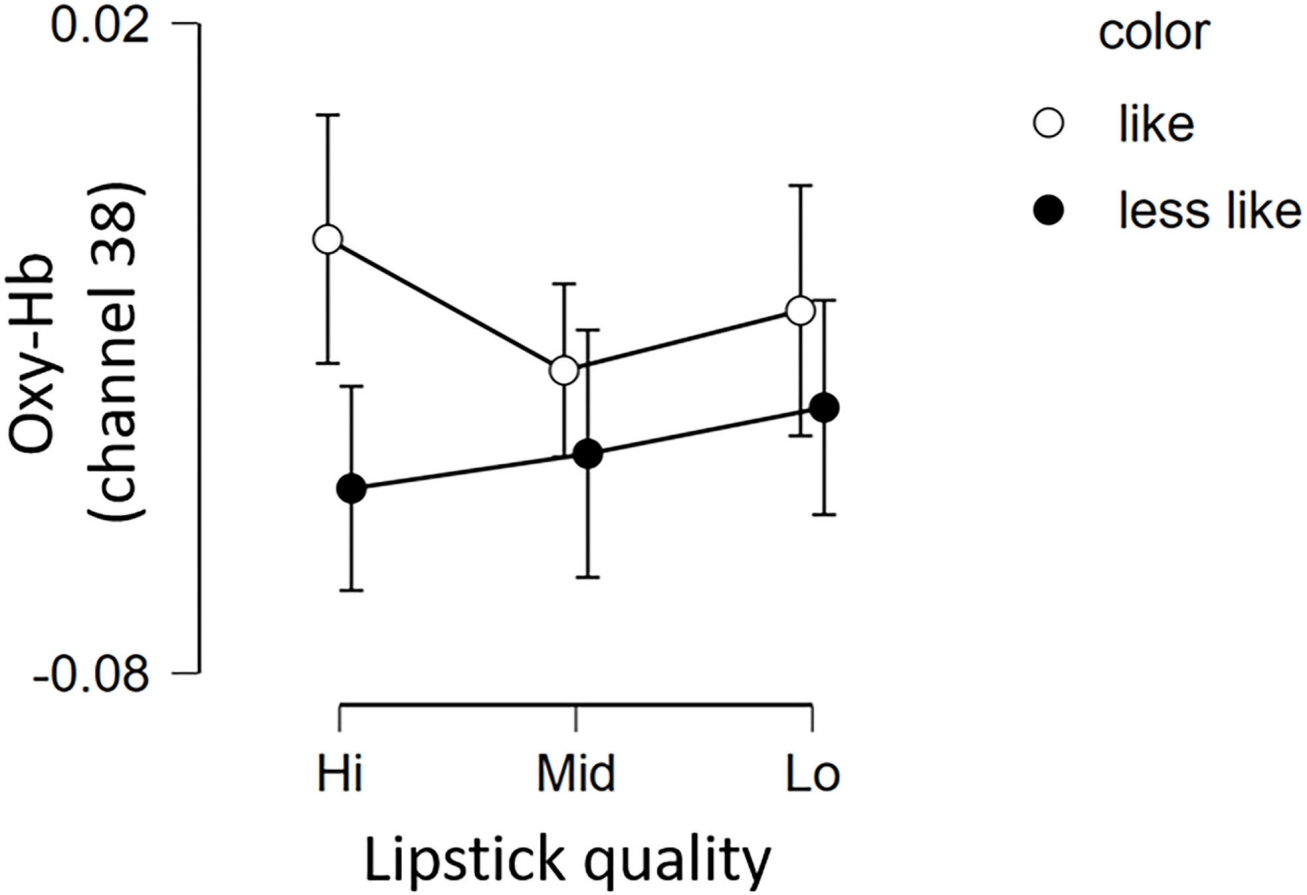
Brain activation in channel 38 for the 6 lipsticks. Graph showing the group average Oxy-Hb of channel 38 of the 3 qualities for the 2 color conditions (*less like* and *like*). Error bars represent the standard error of the mean. Oxy-Hb, oxygenated hemoglobin.

The fNIRS time series date of channel 38 can be seen in Figure 6. First, we investigated whether there was any effect of the different lipsticks on activation in channel 38 (corresponding to the right dlPFC) by using a 2 × 3 repeated-measures ANOVA with color (*like* and *less like*) and as quality (*hi, mid*, and *lo*) as within the factors of subjects. As can be seen in Figure 7, there was no main effect of color [*F*_(1, 24)_ = 2.117, *p* = 0.159, η*p*^2^ = 0.081], no main effect of quality [*F*_(2, 48)_ = 0.180, *p* = 0.836, η*p*^2^ = 0.007], and no interaction [*F*_(2, 48)_ = 0.288, *p* = 0.751, η*p*^2^ = 0.012]. Since the absence of a difference may reflect a lack of statistical power, we also tested whether the brain activity of *less like, lo* quality was different to that of *like, hi* quality. However, there was no evidence of any difference [*like, hi* quality: −0.013, *less like, lo* quality beta: −0.039; *t*_(24)_ = 1.016, *p* = 0.320, *d* = 0.203].

As described above, the coefficient for correction of each subject between their *Apply* period beta values and each WTP score was calculated and then transformed using Fisher's Z transformation into a Z score, to allow group analyses of these intrasubject correlations. Next, a Shapiro-Wilk test was used to check for violations of normality of the Z scores data (Shapiro et al., 1968). The result was statistically insignificant (*p* = 0.879), indicating that there was no evidence of a violation of the assumption of normality. Therefore, one-sample *t-*test was conducted and indicated that the group Z scores for channel 38 differed significantly from 0 (mean Z = 0.239, SEM = 0.091), [*t*_(24)_ = 2.62, *p* = 0.015, *d* = 0.523, see Supplementary Information]. The group R, calculated using the inverse Fisher transformation, was 0.235. The equivalent correlations for oxy-Hb and deoxy-Hb for the remaining channels were not significant (see Supplementary Information for details).

## Discussion

Incorporating neuroscientific measures into product testing may increase the ability of cosmetic product testing to predict the reaction of consumers to the product. As a first step toward this goal, it is critical to determine if meaningful brain activity can be recorded using fNIRS during a single, real use of a cosmetic. Therefore, the first objective of the current study was to replicate the result of the previous study while using a different cosmetic product as a sample. By modifying the design of the previous study, the second objective was to identify consistent differences across subjects between lipsticks.

With respect to the first objective, we found a significant intrasubject correlation between the right dlPFC activity of participant and their WTP. In other words, we could replicate the previous study. This is important because there is concern regarding neuroimaging reproducibility (Poldrack et al., 2020) especially when sample sizes are small (Evans, 2017). The replication is particularly important in the current design because of limitations in how many times the face can be cleansed of cosmetics in a single session. This limitation means that only one trial per cosmetic, six trials in total are possible. We replicated the result in a different population with a different product strongly supports the validity of the findings. In contrast, despite optimizing the previous design, there was no consistent group difference between products in the right dlPFC activity. This is not unexpected, however, given that we were unable to find consistent group differences in WTP for the different lipsticks despite manipulating the color and quality of the lipsticks to maximize their differences. It is unclear why this manipulation did not have the expected effect.

While we replicated our previous finding (Kawabata Duncan et al., 2019), we were unable to detect any consistent group differences of brain activations across six different products. This replicates the results of our previous study. Considering the ratio of observations to variables, this may reflect an insufficient number of participants, however, there was also no difference between brain activity for only the *like, hi* quality and *less like, lo* quality samples. Moreover, despite our expectations, the manipulation of color and quality did not result in consistent group differences in WTPs. It is not clear why *like* and *less like* conditions would not naturally lead to *like* having higher average WTP. One possibility is that the manipulation of both color and quality results in a complex interaction with personal preference. In other words, a *less liked* color of lipstick may increase in liking depending on the quality, because the quality determines how the color will appear on the lips. Limiting the manipulation of lipsticks to only color could potentially resolve this.

The restriction in the number of trials to six is necessary due to the need to minimize the burden of removing cosmetics from the delicate skin of the face and lips. Cleansing is limited to 3 times per session. To increase the number of lipsticks that can be tested, the application area of the lips was divided into left and right sides. The total number of combinations of lipsticks, lip sides, and lip order mean that full counterbalancing is not practical. There remains, therefore, the possibility that the right dlPFC correlation is an artifact of laterality. However, there are two reasons why we think that this is unlikely. First, the PFC functional asymmetry is thought to reflect the left hemispheric involvement for verbal processing and the right hemispheric involvement for nonverbal processing (Opitz et al., 2000; Rothmayr et al., 2007), more than a location in the visual/tactile hemifield. In our study, unguided evaluation of lipstick is likely to rely heavily on non-verbal processes. Second, the current study replicates the result we found in the previous study, which seems unlikely if the current result was an artifact.

However, the underlying reason why there is a correlation between the right dlPFC activity and WTP remains unclear. Without a deeper understanding, it is difficult to predict under what conditions we might expect to find the correlation and when we might not find it. Therefore, investigating this is a critical step before the correlation could be reliably implemented commercially. A further step before implementation is to determine if brain activity is superior in measuring consumer preferences and predicting behavior compared to traditional self-report.

In conclusion, we found an intrasubject correlation, during a single real use of lipstick, between the right dlPFC activities of participants and their WTPs, replicating and extending our previous finding. However, as in the previous study, we were unable to find any consistent group differences in brain activations for different products. This may suggest that the use of the right dlPFC under these conditions may be best suited for a brain-based personalization or product selection process, rather than as a biomarker of all consumer preferences.

## Data Availability Statement

The original contributions presented in the study are included in the article/Supplementary Material, further inquiries can be directed to the corresponding author.

## Ethics Statement

The studies involving human participants were reviewed and approved by Shiseido Co., Ltd. and Chuo University. The patients/participants provided their written informed consent to participate in this study. Written informed consent was obtained from the individual(s) for the publication of any potentially identifiable images or data included in this article.

## Author Contributions

KH, KK, KT, and ID designed the research. KH, KK, TN, and TT performed the experiment. KH, TT, TN, KK, and ID analyzed the data. KH drafted the work. KK and ID revised the manuscript. All authors contributed to the article and approved the submitted version.

## Conflict of Interest

KH, KK, and KT are employed by Shiseido Global Innovation Center, Shiseido Co., Ltd., a company which manufactures cosmetics. KH, TT, KK, KT, and ID are named inventors on a patent application covering the method disclosed in this research. This study received funding from Shiseido Global Innovation Center. The funder was involved in the study design, collection, analysis, interpretation of data, the writing of this article and the decision to submit it for publication. The remaining author declares that the research was conducted in the absence of any commercial or financial relationships that could be construed as a potential conflict of interest.

## Publisher's Note

All claims expressed in this article are solely those of the authors and do not necessarily represent those of their affiliated organizations, or those of the publisher, the editors and the reviewers. Any product that may be evaluated in this article, or claim that may be made by its manufacturer, is not guaranteed or endorsed by the publisher.
