# Supplementary material for: A Willingness-to-Pay Associated Right Prefrontal Activation During a Single, Real Use of Lipsticks as Assessed Using Functional Near-Infrared Spectroscopy
Source: Front Neuroergon. 2021 Nov 26;2:731160. doi: 10.3389/fnrgo.2021.731160 (PMC10790833; doi:10.3389/fnrgo.2021.731160)
Supplement: Supplementary file 1 [file Data_Sheet_1.PDF]

Supplementary Material

A Willingness-to-Pay Associated Right Prefrontal Activation During a Single, Real Use of lipsticks as assessed using Functional Near-Infrared Spectroscopy

Kazue Hirabayashi[1*], Tatsuya Tokuda[2], Tomomi Nishinuma[2], Keith Kawabata Duncan[1], Keiko Tagai[1], Ippeita Dan[2]

[1]MIRAI Technology Institute, Shiseido Co., Ltd., Yokohama, Japan
[2]Applied Cognitive Neuroscience Laboratory, Chuo University, Tokyo, Japan

*** Correspondence:**
Kazue Hirabayashi

kazue.hirabayashi@shiseido.com

Internal

1 Supplementary information

Table S1: Results of one sample t test of oxy-Hb.

Channel	t	p	ES	MeanR
1	0.74	0.47	0.15	0.07
2	-0.84	0.41	-0.17	-0.06
3	1.82	0.08	0.36	0.18
4	0.81	0.42	0.16	0.11
5	-0.50	0.62	-0.10	-0.06
6	-0.54	0.59	-0.11	-0.07
7	-0.63	0.53	-0.13	-0.07
8	-0.59	0.56	-0.12	-0.06
9	0.57	0.58	0.11	0.08
10	0.19	0.85	0.04	0.02
11	1.38	0.18	0.28	0.13
12	-2.04	0.05	-0.41	-0.14
13	0.35	0.73	0.07	0.05
14	0.80	0.43	0.16	0.10

Internal

**15**	**0.56**	**0.58**	**0.11**	**0.08**
**16**	**0.16**	**0.87**	**0.03**	**0.02**
**17**	**-1.13**	**0.27**	**-0.23**	**-0.16**
**18**	**-0.35**	**0.73**	**-0.07**	**-0.03**
**19**	**-0.26**	**0.80**	**-0.05**	**-0.03**
**20**	**0.56**	**0.58**	**0.11**	**0.07**
**21**	**-0.45**	**0.66**	**-0.09**	**-0.05**
**22**	**-0.85**	**0.40**	**-0.17**	**-0.10**
**23**	**-0.06**	**0.96**	**-0.01**	**-0.01**
**24**	**-0.07**	**0.94**	**-0.01**	**-0.01**
**25**	**1.13**	**0.27**	**0.23**	**0.11**
**26**	**0.86**	**0.40**	**0.17**	**0.06**
**27**	**NaN**	**NaN**	**NaN**	**NaN**
**28**	**1.31**	**0.20**	**0.26**	**0.14**
**29**	**-1.08**	**0.29**	**-0.22**	**-0.11**
**30**	**0.79**	**0.44**	**0.16**	**0.10**
**31**	**0.74**	**0.47**	**0.15**	**0.08**

Internal

**32**	**0.62**	**0.54**	**0.12**	**0.07**
**33**	**0.26**	**0.80**	**0.05**	**0.02**
**34**	**-0.15**	**0.88**	**-0.03**	**-0.02**
**35**	**1.98**	**0.06**	**0.40**	**0.19**
**36**	**0.93**	**0.36**	**0.19**	**0.10**
**37**	**0.53**	**0.60**	**0.11**	**0.06**
**38**	**2.62**	**0.02**	**0.52**	**0.23**
**39**	**0.46**	**0.65**	**0.09**	**0.04**
**40**	**-0.48**	**0.64**	**-0.10**	**-0.04**
**41**	**0.27**	**0.79**	**0.05**	**0.03**
**42**	**0.30**	**0.77**	**0.06**	**0.04**
**43**	**-0.49**	**0.63**	**-0.10**	**-0.05**
**44**	**0.89**	**0.38**	**0.18**	**0.10**
**45**	**1.27**	**0.22**	**0.25**	**0.14**
**46**	**0.13**	**0.89**	**0.03**	**0.02**
**47**	**0.17**	**0.87**	**0.03**	**0.02**
**48**	**1.71**	**0.10**	**0.34**	**0.21**

Internal

**49**	**1.43**	**0.17**	**0.29**	**0.13**
**50**	**0.86**	**0.40**	**0.17**	**0.08**
**51**	**1.44**	**0.16**	**0.29**	**0.22**
**52**	**0.56**	**0.58**	**0.11**	**0.06**

Table S2: Results of one sample t test of deoxy-Hb.

Channel	t	p	ES	MeanR
**1**	**-0.69**	**0.50**	**-0.14**	**-0.08**
**2**	**0.28**	**0.78**	**0.06**	**0.03**
**3**	**1.04**	**0.31**	**0.21**	**0.14**
**4**	**-2.20**	**0.04**	**-0.44**	**-0.22**
**5**	**-1.88**	**0.07**	**-0.38**	**-0.18**
**6**	**-0.50**	**0.62**	**-0.10**	**-0.07**
**7**	**0.14**	**0.89**	**0.03**	**0.01**
**8**	**-0.09**	**0.93**	**-0.02**	**-0.01**
**9**	**0.19**	**0.85**	**0.04**	**0.02**
**10**	**0.70**	**0.49**	**0.14**	**0.05**
**11**	**0.55**	**0.59**	**0.11**	**0.07**

Internal

**12**	**-0.01**	**0.99**	**0.00**	**0.00**
**13**	**-0.17**	**0.87**	**-0.03**	**-0.02**
**14**	**-0.35**	**0.73**	**-0.07**	**-0.05**
**15**	**-0.97**	**0.34**	**-0.19**	**-0.12**
**16**	**-0.56**	**0.58**	**-0.11**	**-0.07**
**17**	**0.20**	**0.84**	**0.04**	**0.03**
**18**	**0.31**	**0.76**	**0.06**	**0.04**
**19**	**0.87**	**0.39**	**0.17**	**0.07**
**20**	**1.55**	**0.13**	**0.31**	**0.16**
**21**	**-0.02**	**0.99**	**0.00**	**0.00**
**22**	**-0.71**	**0.49**	**-0.14**	**-0.06**
**23**	**0.51**	**0.62**	**0.10**	**0.06**
**24**	**-0.24**	**0.81**	**-0.05**	**-0.03**
**25**	**0.35**	**0.73**	**0.07**	**0.04**
**26**	**-0.55**	**0.59**	**-0.11**	**-0.06**
**27**	**0.75**	**0.46**	**0.15**	**0.08**
**28**	**-1.38**	**0.18**	**-0.28**	**-0.15**

Internal

**29**	**-0.93**	**0.36**	**-0.19**	**-0.10**
**30**	**1.10**	**0.28**	**0.22**	**0.11**
**31**	**0.32**	**0.75**	**0.06**	**0.03**
**32**	**-1.14**	**0.27**	**-0.23**	**-0.11**
**33**	**0.33**	**0.75**	**0.07**	**0.04**
**34**	**-0.22**	**0.83**	**-0.04**	**-0.03**
**35**	**-1.43**	**0.16**	**-0.29**	**-0.15**
**36**	**1.16**	**0.26**	**0.23**	**0.13**
**37**	**0.12**	**0.90**	**0.02**	**0.01**
**38**	**0.70**	**0.49**	**0.14**	**0.08**
**39**	**0.65**	**0.52**	**0.13**	**0.08**
**40**	**0.38**	**0.70**	**0.08**	**0.04**
**41**	**0.10**	**0.92**	**0.02**	**0.01**
**42**	**-1.26**	**0.22**	**-0.25**	**-0.17**
**43**	**-1.93**	**0.07**	**-0.39**	**-0.20**
**44**	**1.96**	**0.06**	**0.39**	**0.18**
**45**	**1.86**	**0.08**	**0.37**	**0.18**

Internal

**46**	**1.15**	**0.26**	**0.23**	**0.10**
**47**	**0.64**	**0.53**	**0.13**	**0.06**
**48**	**1.00**	**0.33**	**0.20**	**0.12**
**49**	**0.32**	**0.75**	**0.06**	**0.04**
**50**	**-1.26**	**0.22**	**-0.25**	**-0.19**
**51**	**-1.25**	**0.23**	**-0.25**	**-0.12**
**52**	**0.37**	**0.71**	**0.07**	**0.04**

1.1 fNIRS results

Our hypothesis of the relationship between WTP and a channel covering R-dlPFC. Based on the MNI coordinates, ch38 was the area of R-dlPFC and the only channel shows the positive significant correlation with WTP (See in Table S1 and Table S2).

Internal
